# Evaluation of marketing authorization and labels of medicines in 2021 WHO Model List of Essential Medicines for Children in China, the Russian Federation and Brazil

**DOI:** 10.1186/s12961-024-01117-7

**Published:** 2024-03-05

**Authors:** Caiyun Li, Luyan Cheng, Xuefang Zhang, Lingli Zhang, Jianzhou Yan

**Affiliations:** 1 School of International Pharmaceutical Business, China Pharmaceutical University, No. 639 Longmian Avenue, Jiangning District, Nanjing, 211198 China; 2 The Research Center of National Drug Policy and Ecosystem, China Pharmaceutical University, No. 639 Longmian Avenue, Jiangning District, Nanjing, 211198 China; 3 School of Pharmacy, Nanjing Medical University, No. 101 Longmian Avenue, Jiangning District, Nanjing, 211166 China

**Keywords:** Essential medicines for children, Drug labels, China, The Russian Federation, Brazil

## Abstract

**Objective:**

This work compares the marketing authorization, labels and dosage forms of medicines in the WHO Model List of Essential Medicines for Children (EMLc) in China, the Russian Federation and Brazil to urge policymakers to pay more attention to paediatric medication.

**Methods:**

Medicines were selected from the 8th EMLc. By searching relevant databases, which include different types of medical information in China, the Russian Federation and Brazil, the marketing authorization, labels and dosage forms of paediatric medicines in the three countries were evaluated.

**Results:**

A total of 485 drug products containing 312 active pharmaceutical ingredients listed in the WHO EMLc were evaluated. Among them, 344 products were approved for use in China, 286 in the Russian Federation and 264 in Brazil. Out of the 344 approved medicines, 317 (92.15%) were authorized for paediatric use in China, 224 (78.32%) in the Russian Federation and 218 (82.58%) in Brazil. In terms of guidance information labelling on drug labels, 75.08%, 83.04% and 88.07% of paediatric drugs approved in China, the Russian Federation and Brazil, respectively, clearly indicated the usage and dosage for paediatric use. Additionally, injections and tablets were the most prevalent dosage forms in these three countries.

**Conclusion:**

There is still scope for enhancing the marketing authorization and development of dosage forms for paediatric medicines in the three countries. Furthermore, additional measures are being implemented to enhance the information provided on drug labels for children, particularly in China.

**Supplementary Information:**

The online version contains supplementary material available at 10.1186/s12961-024-01117-7.

## Introduction

Children make up a large proportion of the global population, and their physiological and pharmacokinetic traits determine the unique nature of medication for them [1, 2]. To ensure the safe use of drugs for children, relevant national departments of many countries have issued policy documents, encouraging pharmaceutical enterprises to pay more attention to research and development and production of drug products for paediatric patients [3–6]. The approval of medicines for paediatric patients has increased due to support from national policies and favourable industrial policies. However, due to the lower prevalence in children compared with adults, the challenges of involving children in clinical trials, the high costs associated with conducting drug research and development for children and the reduced commercial incentive, pharmaceutical companies have been reluctant to invest in developing specific treatments or adapting existing medicines to cater the needs of the paediatric population [7, 8]. This led to medicines being inaccessible to children, as well as a lack of age-appropriate formulations, adequate dosing and administrations information in the product labels [9–14].

Essential medicines are defined as medicines that satisfy the healthcare needs of the population. In 1977, the WHO adopted and initiated the implementation of the first edition of the Model List of Essential Medicines (EML). It also guided the member countries to develop their national list of essential medicines and implement the system of essential medicines to guarantee basic drug use according to the needs of the public, safeguard the right of citizens to life and health and promote rational clinical medication [15]. Over the past 40 years, the WHO has revised the model list of essential medicines every 2 years on average on the basis of the global burden of diseases, the comparative effectiveness, safety, cost-effectiveness, potency and public health need of the medicines [16]. To promote research and development of paediatric drugs, improve the current situation of irrational usage of paediatric medication, enhance access to essential medicines for children and make countries pay more attention to the effectiveness, safety and economy of paediatric drugs, a resolution on Better Medicine for Children (WHA60.20) was put forward and passed at the World Health Assembly in May 2007 [17]. As the resolution was passed, the WHO set up an expert subcommittee to draft the list of essential medicines for children, and published the first edition of the WHO Model List of Essential Medicines for Children (EMLc) (2007) on 25 October, which included relatively safe, effective, economical and available medicines. The WHO EMLc provides a priority list of medicines for paediatric healthcare requirements. Since its promulgation in 2007, the EMLc has been adjusted and revised every 2 years. The latest edition (the 9th edition) was updated in 2023 [18].

Over the past few decades, Brazil, the Russian Federation, India, China and South Africa (the BRICS countries) have occupied a unique position in the world and in the international health community. Their growing economies have lifted hundreds of millions out of poverty and marked improvements have also taken place in health outcomes [19]. The BRICS countries have developed essential medicine lists. India and South Africa have developed essential medicine lists for children, whereas China, the Russian Federation and Brazil have not.

To enhance the level of drug use guarantee, the concept of essential medicines was introduced in China in 1979, and the first edition of National Essential Medicines List (NEML) was issued in 1982. China has revised and promulgated nine editions of the NEML, and the most recent edition was issued in 2018. [20]. However, on the basis of the findings from the seventh census of China in 2020 and the 2020 Chinese Health Statistics Yearbook [21, 22], challenges persist in ensuring paediatric medication access, including limited availability of child-appropriate medicines with dosage specifications, significant deficiency in dosage form standards and absence of dosing guidance on paediatric drug labels. This has resulted in irrational medication, including a lack of paediatric medicines, substitution with medicines for adults and over-the-counter medication. Brazil has maintained a National Essential Medicines List (EML) since 1964, which has guided health systems to adopt rational therapeutic choices of medicines in clinical practice [23]. However, the pharmaceutical industry and public and private research centres have not invested sufficiently in improving technologies for paediatric care or developing new drugs. This situation makes the use of medication less safe, making it difficult to predict and reliably treat therapeutic outcomes in the paediatric population [24, 25]. To provide free medicines to the citizens by the Ministry of Health, the structure of the morbidity was analysed and the Vital and Essential Drug list (VEDL) was compiled to ensure that priority healthcare needs for the prevention and treatment of main diseases in the structure of the morbidity are taken care of in the Russian Federation. This list is subject to approval by the government annually [26]. However, many medicines are not approved for paediatric use in Russian Federation [27].

Hence, it is of great significance to study the guarantee level of paediatric medication in three BRICS countries without an essential medicine list for children (Table 1). This issue has not been explored by other scholars. Through analysing the registered number of approvals, labels and dosage forms of medicines in WHO EMLc, this study aimed to evaluate levels of marketing authorization and paediatric medication information on the labels and dosage forms of paediatric drugs in China, the Russian Federation and Brazil. It also aims to provide a reference to facilitate administration and acceptance in the paediatric population.

**Table 1:** Health observatory of China, the Russian Federation and Brazil

Country	China	The Russian Federation	Brazil
Income level	Upper-middle income	Upper-middle income	Upper-middle income
Gross national income per capita (Intl $) (2019)	16 610	29 120	14 890
Total expenditure on health per capita (Intl $) (2019)	880.19	1704.04	1497.81
Total expenditure on health as % of GDP (2019)	5.35%	5.65%	9.59%
Population ages 0–14 (thousands)	254 352.48	25 472.81	44 418.96
Population between ages of 0 and 14 years as a percentage of the total population	18%	18%	21%
Essential Medicines List (last edition)	2018	2023	2020
Essential Medicines List for Children	NA	NA	NA

Source: The World Bank

*NA* the country has not developed the Essential Medicines List for children

## Material and methods

### Material

The 8th WHO EMLc was chosen as the focus of our research, and we translated and summarized the active ingredients, specifications and dosage forms of the listed medicines [28]. To enhance the feasibility of the research, medicines and vaccines for which the effective active ingredients and dosage forms could not be determined were excluded (Table 2). In this study, active ingredients referred to compounds with real pharmacological activity in medicines, and drug products referred to the active ingredient combined with a specific dosage form of a drug. In conclusion, 485 drug products (312 active ingredients) were assessed. The paediatric age range considered was 0–12 years, aligning with the age range specified in EMLc.

**Table 2:** Medicines excluded from the study

Number	Medicines in EMLc	Number	Medicines in EMLc
1	Fresh-frozen plasma	20	Tetanus vaccine
2	Platelets	21	Japanese encephalitis vaccine
3	Red blood cells	22	Tick-borne encephalitis vaccine
4	Whole blood	23	Yellow fever vaccine
5	Normal immunoglobulin	24	Cholera vaccine
6	Alcohol-based hand rub	25	Dengue vaccine
7	Chlorine base compound	26	Hepatitis A vaccine
8	Oral rehydration salts	27	Meningococcal meningitis vaccine
9	BCG vaccine	28	Rabies vaccine
10	Diphtheria vaccine	29	Typhoid vaccine
11	Haemophilus influenzae type b vaccine	30	Influenza vaccine (seasonal)
12	Hepatitis B vaccine	31	Mumps vaccine
13	Human papilloma virus (HPV) vaccine	32	Varicella vaccine
14	Measles vaccine	33	Surfactant
15	Pertussis vaccine	34	Intraperitoneal dialysis solution
16	Pneumococcal vaccine	35	Sodium lactate, compound solution
17	Poliomyelitis vaccine	36	Water for injection
18	Rotavirus vaccine	37	Multiple micronutrient powder
19	Rubella vaccine	38	Fluoride

### Data extraction and collection

Registered number of approvals, and sections such as dosage forms, indication, posology and method of administration, contraindications, warnings and precautions, summary of clinical trials and pharmacokinetics in special population—children, on drug labels were extracted and collected. On the basis of the above information, we have sorted out the following key points: (1) approval status of the drug product, (2) whether the drug product approved for paediatric use, (3) paediatric medication information on drug labels and (4) dosage forms. A drug product is considered to be approved for use in the country if its registered number is active. A drug product is considered appropriate for use in children if it meets one of the following conditions: (1) the indications for use in children or the dosage for use in children are clearly indicated in the drug labels, (2) the indications, usage and dosage do not specify the drug group (adults, the elderly, children, infants, etc.), and there are no prohibitions for children in the notes for attention. The level of paediatric medication information is determined according to the information provided in the drug labels. Those with clear usage and dosage in the drug labels are considered active.

Registered numbers of drugs were collected from China National Medical Products Administration (https://www.nmpa.gov.cn/datasearch/home-index.html?3jfdxVGGVXFo=1684828499150#category=yp), the Russian National Drug Degustation (https://grls.rosminzdrav.ru/grls.aspx?s=ибyпpoфeн&m=mnn) and the Brazilian National Health Surveillance Agency (https://consultas.anvisa.gov.br/#/medicamentos/). Drug labels were collected from Yaozhi database (https://db.yaozh.com/instruct), the Russian National Drug Degustation and the Brazilian National Health Surveillance Agency. Portuguese and Russian were translated into English using Google Translate. The materials of the drug products were collected in June of 2022. All collections were conducted independently by three separate reviewers (CL, LC and XZ), and if the result of collections was inconsistent, the three reviewers searched together and determined the results.

### Data analysis

In the previous research on paediatric medication, percentages were commonly used to indicate the level of paediatric medication security. Therefore, to intuitively analyse the supply guarantee level of paediatric drugs in the 8th WHO EMLc of three countries, this study refers to available research and describes the current status of paediatric drug guarantee in different countries quantitatively [7]. The percentages of several analyses were calculated using the following equations:

$${\text{Market}}\;{\text{authorization}}\;\% = \left( {{\text{drugs}}\;{\text{approved}}\;{\text{for}}\;{\text{use}}} \right)/\left( {{\text{selected}}\;{\text{drugs}}} \right) \times {1}00\% ,$$

$${\text{Authorization}}\;{\text{of}}\;{\text{pediatric}}\;{\text{medication}}\;\% = \left( {{\text{drugs}}\;{\text{approved}}\;{\text{for}}\;{\text{pediatric}}\;{\text{use}}} \right)/\left( {{\text{drugs}}\;{\text{approved}}\;{\text{for}}\;{\text{use}}} \right) \times {1}00\% ,$$

$${\text{Pediatric}}\;{\text{medication}}\;{\text{information}}\;{\text{in}}\;{\text{the}}\;{\text{labels}}\;\% = \left( {{\text{Drugs}}\;{\text{marked}}\;{\text{with}}\;{\text{detailed}}\;{\text{usage}}\;{\text{and}}\;{\text{dosage}}} \right)/\left( {{\text{drugs}}\;{\text{approved}}\;{\text{for}}\;{\text{pediatric}}\;{\text{use}}} \right) \times {1}00\% .$$

## Results

### Market authorization of medicines in WHO EMLc

**Fig. 1:**
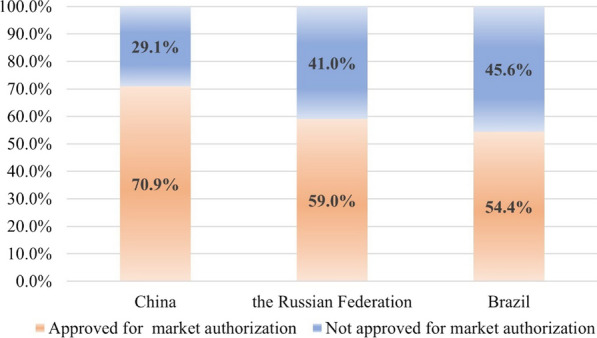
Percentage of medicines in WHO EMLc 2021 listed as approved medicines in China, the Russian Federation and Brazil

**Fig. 2:**
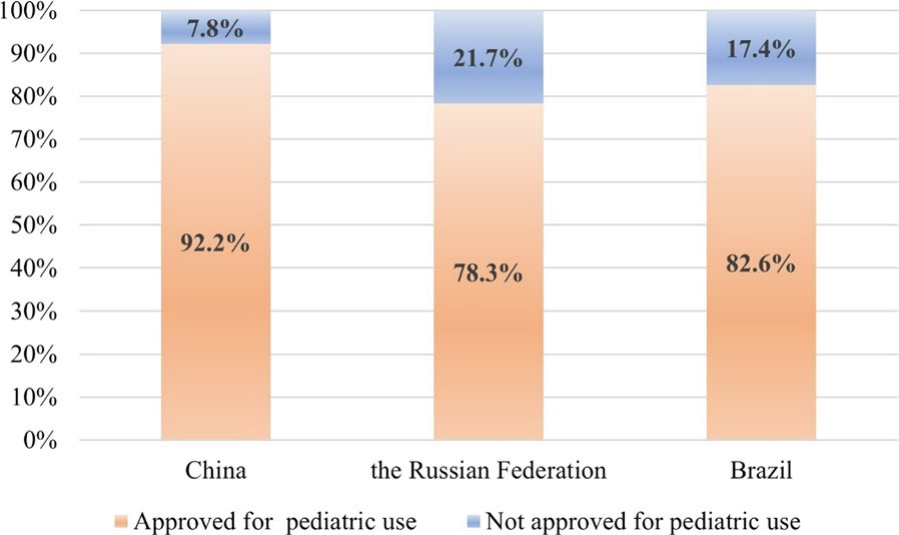
Percentage of medicines approved for use listed as approved for paediatric medication in China, the Russian Federation and Brazil

This study identified 485 drug products. Among them, 344, 286 and 264 have been approved for use in China, the Russian Federation and Brazil, respectively. As illustrated in Fig. 1, the medicines approved for use corresponded to 70.9%, 59.0% and 54.4%. Among the drugs approved for use, 317, 224 and 218 drug products were approved for paediatric use in China, the Russian Federation and Brazil, respectively. The levels of authorization for paediatric medication are displayed in Fig. 2.

The 485 WHO EMLc drug products listed cover 28 disease areas. The 317 drug products approved for children in China covered a total of 27 disease areas, while the 168 drug products not approved for children covered a total of 24 disease areas. The 224 drug products approved for use in children in the Russian Federation cover a total of 25 disease areas, while the 261 drug products not approved for use in children cover a total of 27 disease areas. The 218 drug products approved for children in Brazil covered a total of 24 disease areas, while the 168 drug products not approved for children covered a total of 27 disease areas (Table 3). A complete list of market authorization of Medicines in WHO EMLc in the three countries is available in the Additional file 1: Table S1.

**Table 3:** Comparison of therapeutic areas in WHO EMLc, China, the Russian Federation and Brazil

Therapeutic areas	WHO EMLc	China		The Russian Federation		Brazil	
		Approved for children	Not approved for children	Approved for children	Not approved for children	Approved for children	Not approved for children
Anaesthetics, preoperative medicines and medical gases	17	13	4	9	8	9	8
Medicines for pain and palliative care	31	19	12	13	18	14	17
Anti-allergics and medicines used in anaphylaxis	6	5	1	2	4	5	1
Antidotes and other substances used in poisonings	10	8	2	2	8	0	10
Anticonvulsants/antiepileptics	20	11	9	13	7	11	9
Anti-infective medicines	192	115	77	81	111	90	102
Anti-migraine medicines	1	1	0	0	1	1	0
Immunomodulators and antineoplastics	57	40	17	24	33	21	36
Medicines affecting the blood	13	10	3	9	4	5	8
Blood products of human origin and plasma substitutes	5	4	1	4	1	4	1
Cardiovascular medicines	8	7	1	4	4	4	4
Dermatological medicines (topical)	19	11	8	9	10	8	11
Diagnostic agents	3	2	1	0	3	2	1
Antiseptics and disinfectants	5	4	1	2	3	1	4
Diuretics	5	4	1	2	3	4	1
Gastrointestinal medicines	13	10	3	5	8	4	9
Medicines for endocrine disorders	14	8	6	9	5	5	9
Immunological	6	5	1	4	2	4	2
Muscle relaxants (peripherally acting) and cholinesterase inhibitors	7	5	2	6	1	4	3
Ophthalmological preparations	11	6	5	4	7	2	9
Medicines for reproductive health and perinatal care	4	1	3	1	3	1	3
Medicines for mental and behavioural disorders	6	4	2	3	3	3	3
Medicines acting on the respiratory tract	4	4	0	2	2	3	1
Solutions correcting water, electrolyte and acid–base disturbances	7	5	2	5	2	6	1
Vitamins and minerals	12	9	3	7	5	6	6
Ear, nose and throat medicines	3	3	0	1	2	0	3
Medicines for diseases of joints	3	3	0	3	0	1	2
Dental preparations	3	0	3	0	3	0	3

### Paediatric medication information in the labels

**Fig. 3:**
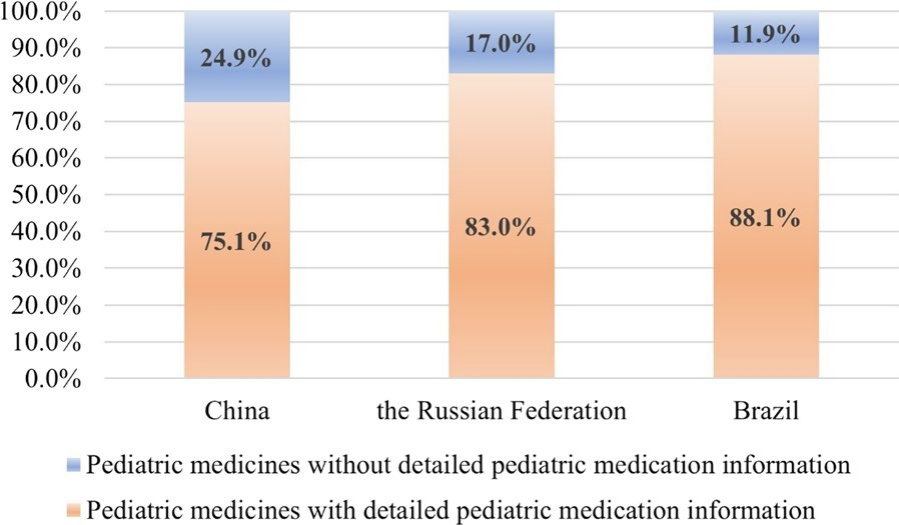
Percentage of medicines approved for a paediatric medication listed with detailed paediatric medication information in instructions

Figure 3 presents the labelling level of drug use guidance information for drug products approved for paediatric medication. Among the 317 drug products approved for paediatric use in China, 238 were clearly labelled with usage and dosage information for paediatric patients. In comparison, 186 and 192 drug products in the Russian Federation and Brazil, respectively, contained the required information. The labelling levels of drug guidance information for paediatric patients in the three countries were 75.1%, 83.0% and 88.1%, respectively. A complete list is available in the Additional file 1: Table S1.

### Administration routes and dosage forms of drug products approved for paediatric patients

The main administration route in WHO EMLc is oral administration, the second most frequent administration route is injection and the third most frequent administration route is topical use. The proportion of the three main administration routes is 55.3%, 32.0% and 7.2%, respectively. The main administration route of medicines approved for children of China, the Russian Federation and Brazil is oral administration. The proportion of oral administration of medicines approved for children of China, the Russian Federation and Brazil is 49.8%, 50.4% and 56.0%, respectively. The second most frequent administration route is injection. The proportion of injection of medicines approved for children of China, the Russian Federation and Brazil is 37.5%, 37.9% and 33.9%, respectively. The third most frequent administration route is topical use. The proportion of topical use of medicines approved for children of China, the Russian Federation and Brazil is 7.3%, 6.3% and 6.0%, respectively (Table 4). A complete list is available in the Additional file 1: Table S1.

**Table 4:** Comparison of administration routes of WHO EMLc and drug products approved for children in China, the Russian Federation and Brazil

	WHO (%)	China (%)	Russia (%)	Brazil (%)
Oral	268 (55.3%)	158 (49.8%)	113 (50.4%)	122 (56.0%)
Injective	155 (32.0%)	119 (37.5%)	85 (37.9%)	74 (33.9%)
Topical	35 (7.2%)	23 (7.3%)	14 (6.3%)	13 (6.0%)
Nasal	9 (1.9%)	8 (2.5%)	4 (1.8%)	6 (2.8%)
Ophthalmic	11 (2.3%)	5 (1.6%)	3 (1.3%)	2 (0.9%)
Rectal	4 (0.8%)	2 (0.6%)	3 (1.3%)	1 (0.5%)
Otological	1 (0.2%)	1 (0.3%)	1 (0.4%)	–
Buccal	2 (0.4%)	1 (0.3%)	1 (0.4%)	–

As Table 5 presents, there are 29 dosage forms in WHO EMLc, amongst which, there are 11 (37.9%) oral dosage forms. There are 24, 22 and 22 dosage forms of the drug products approved for children in China, the Russian Federation, and Brazil, respectively. Compared with WHO EMLc, drug products approved for children in China did not cover granules, rectal dosage form, dental cartridge, infusion and lozenge. Drug products approved for children in the Russian Federation did not cover granules, dental cartridge, infusion, lozenge, tablet (crushable), tablet (sugar-coated) and transdermal patches. Drug products approved for children in Brazil did not cover granules, rectal dosage form, dental cartridge, ear drops, eye ointment, infusion, lozenge, solution for oromucosal administration and transdermal patches. Among these, granules, rectal dosage form, infusion, lozenge, tablet (crushable), tablet (sugar-coated), transdermal patches, ear drops, eye ointment and solution for oromucosal administration are considered to be children-suitable dosage forms.

**Table 5:** Comparison of dosage forms of drug products approved for children in China, the Russian Federation and Brazil

	WHO	China	Russia	Brazil
Injection	155	119	85	74
Tablet	109	80	49	56
Oral liquid	62	20	20	24
Capsule	25	16	9	11
Solid oral dosage form	25	21	17	13
Tablet (dispersible)	18	4	7	5
Cream or ointment	13	10	7	7
Solution	12	8	4	3
Eye drops	10	4	2	2
Powder for oral liquid	10	9	4	6
Inhalation	7	6	3	5
Tablet (chewable)	7	4	5	2
Granules	5	0	0	0
Tablet (scored)	5	2	2	3
Lotion	4	1	1	1
Nasal spray	2	2	1	1
Powder for solution	2	2	1	1
Rectal dosage form	2	0	1	0
Suppository	2	2	2	1
Dental cartridge	1	0	0	0
Ear drops	1	1	1	0
Eye ointment	1	1	1	0
Infusion	1	0	0	0
Lozenge	1	0	0	0
Solution for oromucosal administration	1	1	1	0
Tablet (crushable)	1	1	0	1
Tablet (sugar-coated)	1	1	0	1
Topical forms	1	1	1	1
Transdermal patches	1	1	0	0

## Discussion

This study confirms the necessity for improvements in drug in paediatrics and the lack of age-appropriate medicines in many therapeutic areas. Efforts are underway to enhance the development and approval of drug products tailored for children in response to regulatory demands.

Judging from the marketing level of drug products in the WHO EMLc in the three countries, China currently has the largest number of drug products approved for clinical use. Analysis of the clinical application of the approved drug products revealed that 92.2%, 78.3% and 82.6% drug products were approved for paediatric patients.

However, the WHO EMLc is formulated on the basis of the disease burden and clinical demand of the global paediatric population. Additionally, the disease spectrum and disease burden vary among different countries, so not all listed drugs may be relevant for every clinical settings. On the basis of the data of China, the Russian Federation and Brazil in the Global Burden of Disease Study 2019, we identified the top 25 causes in China, the Russian Federation and Brazil of children under 14 years, ranked by disability-adjusted life years (DALYs) [29]. The three countries share 17 of the top 25 causes, but their rankings of burden of disease differ in terms of clinical need, which impacts the number of approved drugs. Meanwhile, significant variations exist in the child population base (Table 1) across the three countries, potentially impacting the count of approved drugs. Hence, scientific assessment of drug supply levels in different countries should encompass a comprehensive set of indicators. According to the findings of this study, health departments and drug administration should pay more attention to those drugs with higher disease burden but less drug approval for children.

Drug labels are considered a key source document as they provide science-based prescribing information to guide healthcare professionals to prescribe drugs safely and effectively for their approved indications in paediatric patients, as well as assist healthcare professionals in choosing the most appropriate therapy [30, 31]. This study examined the labelling level of medication instruction information for paediatric patients in the three countries. According to the summary of drug labels from China, the Russian Federation and Brazil, the results demonstrated that 238 of the 317 approved drug products for paediatric patients in China clearly indicated the usage and dosage for paediatric patients, accounting for 75.1%. The remaining 79 products only indicated vague drug use information for paediatric patients in the instructions, such as ‘reducing the drug use amount appropriately’, ‘reducing the drug use amount appropriately according to the weight’ and ‘the drug use amount for children is half that for adults’. In contrast, among the 224 drug products approved for paediatric patients in the Russian Federation, 186 products (83.0%) provide explicit indications for paediatric use and dosage in the instructions and 192 products (88.1%) have explicit indications for paediatric use and dosage in the instructions amongst the 218 drug products approved for paediatric patients in Brazil (Fig. 3). Additionally, the study revealed that the information in the drug labels of the Russian Federation and Brazil was more comprehensive and instructive compared with those in China, providing better safeguards for paediatric medication. The Russian Federation provided great detail on the consumption by children of different age groups. In the drug labels, children are subdivided into specific age groups, and the medication consumption is marked according to the physiological characteristics of the different age groups, which is conducive to ensuring reasonable, safe and accurate medication for paediatric patients [32]. Brazilian regulatory agencies provide detailed usage and dosage information on drug labels on the basis of the age and physiological characteristics of children. For instance, ibuprofen suspension is administered to reduce fever and alleviate pain in paediatric patients. Upon comparing the drug labels, it is evident that ibuprofen suspension is indicated for use in children aged 1–12 years in all three countries (Tables 6, 7, 8). Additionally, in Brazil, ibuprofen suspension is approved for infants aged 6–12 months (Table 7), and in the Russian Federation, it is approved for infants aged 3–12 months (Table 8). In comparison with China, the drug labels in Russia and Brazil also specify the maximum dosage for different age and weight groups of children to ensure the safety of paediatric medication. Furthermore, Brazil’s drug label provides dosage recommendations based on the severity of fever and the specific weight of children.

**Table 6:** The drug label of ibuprofen suspension in China

Age (years)	Weight (kg)	Primary dosage (ml)	Frequency of administer medicine
1–3	10–15	4	If the pain or fever persists, the drug can be repeated once every 4–6 h, and no more than four times in 24 h
4–6	16–21	5	
7–9	22–27	8	
10–12	28–32	10

**Table 7:** The drug label of ibuprofen suspension in Brazil

Usage and dosage
The recommended dosage for children older than 6 months is 1–2 drops/kg, with an interval of 6–8 h, 3–4 times a day
The maximum dosage for children under 12 years is 40 drops (200 mg) each time, and the maximum allowable dose is 160 drops (800 mg) a day

Maximum dosage recommended per time, four times a day					
Weight (kg)	Slight fever (< 39℃)	High fever (≥ 39℃)	Weight (kg)	Slight fever (< 39℃)	High fever (≥ 39℃)
5	5 drops	10 drops	23	23 drops	40 drops
6	6 drops	12 drops	24	24 drops	40 drops
7	7 drops	14 drops	25	25 drops	40 drops
8	8 drops	16 drops	26	26 drops	40 drops
9	9 drops	18 drops	27	27 drops	40 drops
10	10 drops	20 drops	28	28 drops	40 drops
11	11 drops	22 drops	29	29 drops	40 drops
12	12 drops	24 drops	30	30 drops	40 drops
13	13 drops	26 drops	31	31 drops	40 drops
14	14 drops	28 drops	32	32 drops	40 drops
15	15 drops	30 drops	33	33 drops	40 drops
16	16 drops	32 drops	34	34 drops	40 drops
17	17 drops	34 drops	35	35 drops	40 drops
18	18 drops	36 drops	36	36 drops	40 drops
19	19 drops	38 drops	37	37 drops	40 drops
20	20 drops	40 drops	38	38 drops	40 drops
21	21 drops	40 drops	39	39 drops	40 drops
22	22 drops	40 drops	40	40 drops	40 drops

**Table 8:** The drug label of ibuprofen suspension in the Russian Federation

Age	Weight (kg)	Primary dosage (ml)	Frequency of administer medicine (24 h)	Maximum dosage recommended a day
3–6 months	5–7.6	2.5 ml (50 mg)	3	7.5 ml (150 mg)
6–12 months	7.7–9	2.5 ml (50 mg)	3–4	10 ml (200 mg)
1–3 years	10–16	5.0 ml (100 mg)	3	15 ml (300 mg)
4-6 years	17–20	7.5 ml (150 mg)	3	22.5 ml (450 mg)
7-9 years	21–30	10.0 ml (20 mg)	3	30 ml (600 mg)
10–12 years	31–40	15.0 ml (300 mg)	3	45 ml (900 mg)

^*^Ibuprofen is used as an antipyretic for no more than 3 days and as an analgesic for no more than 5 days. Do not exceed the indicated dose

The information provided on the drug labels may vary depending on the country in which the drug is marketed, as a result of differing requirements from regulatory authorities and their approaches to paediatric risk–benefit analysis [33]. The absence of paediatric clinical trials has resulted in limited or no documentation for paediatric use of many approved drugs, leading to insufficient information on drug labels commonly prescribed for children. A cross-sectional observational study of paediatric trials registered in the Drug Trial Registration and Information Publication Platform from 2013 to 2021 displayed that there were 588 registered paediatric clinical trials in China, which accounted for 3.9% of the total registered trials [34]. While 638 in Brazil, there were 833 registered paediatric clinical trials, accounting for 17.9% of the total registered trials (https://clinicaltrials.gov/). Therefore, the level of paediatric medication information on the labels in Brazil surpassed that of China. To ensure patient safety, Russian legislation specifies the conduct of clinical trials involving minors. The regulations mandate consideration of age-specific pharmacokinetics when developing and conducting clinical trials for medications involving minors, leading to more detailed medication information for children in the Russian Federation [35].

The dosage form is crucial for rational paediatric medication, and the absence of an appropriate dosage form for children often affects the safety of the drug use as well as the efficacy and bioavailability of the administered drug [36–38].

In the drug products evaluated in this study, the drug dosage forms were widely distributed, including injection, tablet, oral liquid, capsule, tablet (dispersible), cream, solution, eye drops, powder for oral liquid, inhalation, chewable tablet, granules scored tablet, lotion, nasal spray, powder for solution, rectal dosage form, suppository, dental cartridge, ear drops, eye ointment, infusion, lozenge, solution for oromucosal administration, crushable tablet, sugar-coated tablet, topical forms, transdermal patches and solid oral dosage form. Regarding the distribution of dosage forms of drug products approved for children (Table 5), injection and tablets were the most common dosage forms in the three countries, while oral solutions, granules and other dosage forms considered suitable for children accounted for a relatively small proportion [39]. Therefore, the dosage forms for children warrant development, and new dosage forms suitable for children, such as mini tablets, chewable tablets, sprays and orally soluble films, should be developed by pharmaceutical enterprises [40, 41].

Therefore, all three countries need to pay more attention to the development of paediatric medication. First of all, health departments should identify the areas of paediatric diseases in which drugs are lacking, and relevant departments should take further incentive measures, such as tax exemptions and extension of patent periods, to encourage drug manufacturers to increase the research and development of paediatric drugs. As for improving the instruction information on the paediatric labels, health departments can refer to the practices of the United States and the European Union to strengthen paediatric legislation to improve and modify paediatric labels during the paediatric drug development process and post-marketing period; making safety assessments through these label changes is an essential component of paediatric drug development [42]. This study reveals that China has the lowest level of children’s drug labelling. China can benefit from the practices in Russia and Brazil. China should enhance the development of clinical trials for children and consider the age-specific pharmacokinetics of the target patient group when conducting clinical trials involving minors to provide more detailed medication information for children. In addition, international collaboration can be pursued to share current information on paediatric labels, investigate issues related to paediatric drugs and share drug safety and effectiveness information in paediatric populations collected through other countries. In terms of dosage forms, dosage forms suitable for children should be developed to facilitate administration and acceptance in the paediatric subset. Research suggests that when in severe acute situations, the injectable presentation can be one of choice of dosage form, as it allows for greater precision in the dose administered to children [43]. In the future, pharmaceutical companies should focus on developing mini tablets, sugar-coated tablets, granules, lozenges, oral solutions and other dosage forms suitable for children.

However, there are some limitations that need to be mentioned. Firstly, only drugs of the 8th EMLc developed by the WHO were analysed, but there are considerably more drugs to analyse that are approved for use in the three countries, which could bias the result. Secondly, the research object of our study is the 8th WHO EMLc, not the 9th EMLc, which was updated in July 2023. Thirdly, our study provides a snapshot of the real-word situation, which may change over time and will not fully reflect all the dynamic factors related to authorization availability. Fourthly, due to the constraints of research space, detailed investigations into relevant national policies will be conducted in subsequent studies.

## Conclusion

This quantitative evaluation confirms the necessity for enhancing age-appropriate medications, paediatric drug dosage forms and drug label information to align with paediatric oral biopharmaceutics and capabilities.

### Supplementary Information

**Additional file 1: Table S1. **Evaluation of Pediatric Medication in China, Russia and Brazil for Medicines Contained in the WHO Model List of Essential Medicines for Children 2021.

## Data Availability

The data supporting the findings of this study are available within the Additional file 1: Table S1.
